# Leptomeningeal isolated infiltration in plasma cell dyscrasia associated to HIV

**DOI:** 10.1055/s-0042-1758391

**Published:** 2022-12-29

**Authors:** Flávia Sprenger, Lucas Shibayama, Bernardo Corrêa de Almeida Teixeira

**Affiliations:** 1 Universidade Federal do Paraná, Hospital de Clínicas, Departamento de Radiologia, Curitiba PR, Brazil.

**Figure 1:**
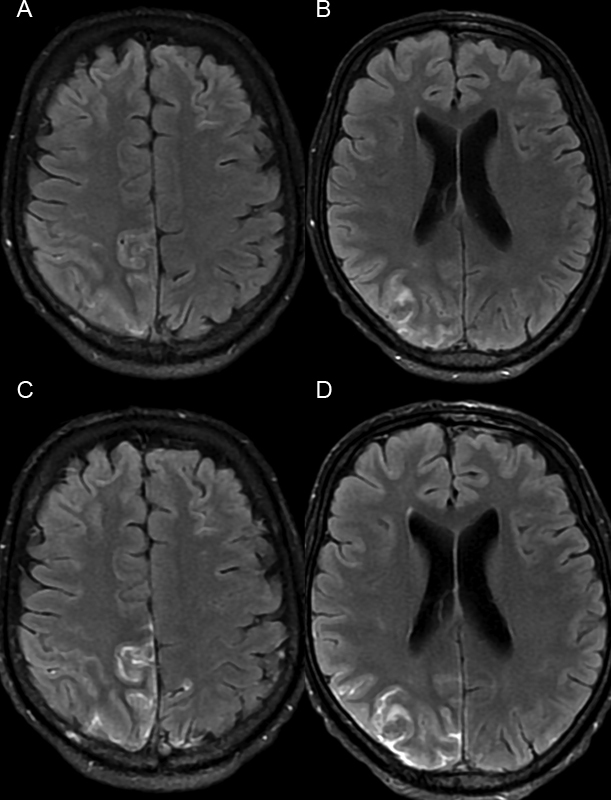
( **A-B** ) Precontrast fluid-attenuated inversion recovery (FLAIR) axial images showing a hyperintensity and thickening of the sulci on the right frontoparietal transition and parietal lobe. ( **C-D)** Postcontrast FLAIR axial images better depicting intense, thick, and irregular leptomeningeal enhancement on the aforementioned regions.

A 52-year-old HIV-positive man (CD4 = 74 cells) presented with amaurosis and headache. The cerebrospinal fluid (CSF) had increased opening pressure and the magnetic resonance imaging (MRI) findings included irregular leptomeningeal thickening on the right frontoparietal transition and parietal sulci, with restricted diffusion, and irregular nodular gadolinium enhancement ( Figures 1 2 3 ). Through CSF immunophenotyping, the final diagnosis of plasma cell dyscrasia with leptomeningeal infiltration was confirmed. HIV is a known risk factor for a wide range of plasma cell dyscrasia, from benign manifestations to aggressive multiple myeloma. 1 Meningeal involvement in multiple myeloma and plasma cell dyscrasias is extremely rare, with less than 70 reported cases. 2

**Figure 2:**
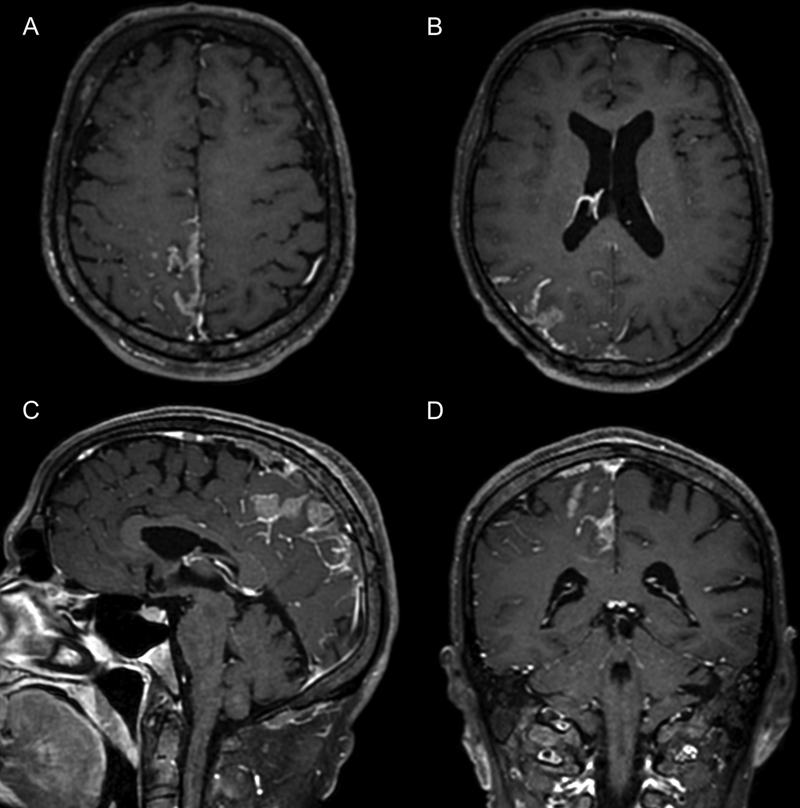
( **A-B** ) Postgadolinium volumetric fast-spin echo black-blood T1-weighted image demonstrating thick and irregular leptomeningeal enhancement on the right frontoparietal and parietal regions. ( **C-D** ) Sagittal and coronal postcontrast vessel wall imaging respectively demonstrating a nodular lesion in the same regions.

**Figure 3:**
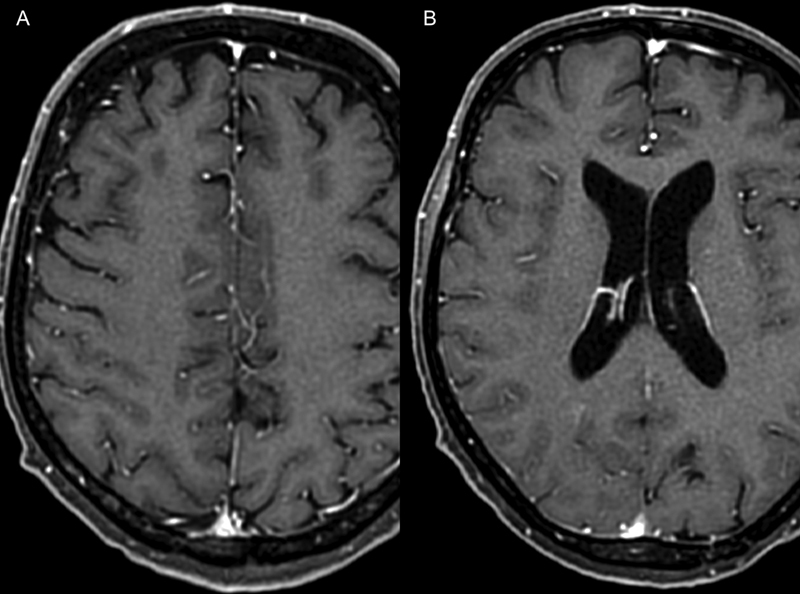
( **A-B** ) Axial T1-weighted image after 35 days of chemotherapy showing complete regression of the leptomeningeal thickening and enhancement.
